# Prognostic Role of Functional Neuroimaging after Multilobar Resection in Patients with Localization-Related Epilepsy

**DOI:** 10.1371/journal.pone.0136565

**Published:** 2015-08-25

**Authors:** Eun Bin Cho, Eun Yeon Joo, Dae-Won Seo, Seung-Chyul Hong, Seung Bong Hong

**Affiliations:** 1 Department of Neurology, Samsung Medical Center, Samsung Biomedical Research Institute, Sungkyunkwan University School of Medicine, Seoul, South Korea; 2 Department of Neurosurgery, Samsung Medical Center, Samsung Biomedical Research Institute, Sungkyunkwan University School of Medicine, Seoul, South Korea; 3 Neuroscience center, Samsung Medical Center, Seoul, South Korea; University of Toronto, CANADA

## Abstract

To investigate the usage of functional neuroimaging as a prognostic tool for seizure recurrence and long-term outcomes in patients with multilobar resection, we recruited 90 patients who received multilobar resections between 1995 and 2013 with at least 1-year follow-up (mean 8.0 years). All patients were monitored using intracranial electroencephalography (EEG) after pre-surgical evaluation. Clinical data (demographics, electrophysiology, and neuroimaging) were reviewed retrospectively. Surgical outcomes were evaluated at 1, 2, 5 years after surgery, and at the end of the study. After 1 year, 56 patients (62.2%) became Engel class I and at the last follow-up, 47 patients (52.2%) remained seizure-free. Furthermore, non-localized ^18^F-fluorodeoxyglucose positron emission tomography (PET), identifying hypometabolic areas not concordant with ictal onset zones, significantly correlated with seizure recurrence after 1 year. Non-lesional magnetic resonance imaging (MRI) and left-sided resection correlated with poor outcomes. In the last follow-up, non-localized PET and left-sided resection significantly correlated with seizure recurrence. Both localized PET and ictal-interictal SPECT subtraction co-registered to MR (SISCOM) predicted good surgical outcomes in the last follow-up (69.2%, Engel I). This study suggests that PET and SISCOM may predict postoperative outcomes for patients after multilobar epilepsy and shows comparable long-term surgical outcomes after multilobar resection.

## Introduction

Medically intractable localization-related epilepsy (LRE) patients have potential to eliminate seizures by resection surgery for epilepsy. Multilobar resection is used for patients with multiple epileptogenic foci. However, surgical outcomes for multilobar resection are worse than single lobe resections.[1–4] Therefore, multilobar resection is regarded to be a poor prognostic factor of surgical outcomes in patients with focal cortical dysplasia [1] and extra-temporal lobe epilepsy.[5,6] Previous studies have reported that 41% of patients undergoing multilobar resection were seizure-free after 10 years.[7] A recent study also showed even better results where 60.6% of patients with two or more lobe resections (54/89) had good outcomes (Engel I-III).[3] However, the duration of follow-up was short (2 years) and the range of ‘good outcomes’ was broader than other reports.

Multiregional or extensive epileptic foci in patients with multilobar epilepsy may lead to incomplete resection and poor surgical outcomes. To achieve good surgical outcomes, comprehensive preoperative evaluation utilizing various diagnostic tools is used. The utility of functional neuroimaging (^18^F-fluorodeoxyglucose positron emission tomography [FDG-PET] and single-photon emission computed tomography [SPECT]) has been demonstrated in several studies.[8–15] However, the results are confined to specific syndromes, such as focal cortical dysplasia,[8,13] non-lesional epilepsy,[11,13–15] frontal, or extra-temporal lobe epilepsy,[9,11,15] and with the small number of study participant.

There have been few studies investigating factors predicting seizure outcomes following multilobar resection.[6,7] In this study, functional neuroimaging data were used to estimate postoperative seizure outcomes. Also, compared to prior studies, the follow-up period was substantially longer (mean 8.0 years, 1–19). We aimed to investigate the usage of functional neuroimaging as prognostic tools for seizure recurrence and long-term seizure outcomes in patients undergoing multilobar resection.

## Methods

### Participants

The study was conducted at Samsung Medical Center, Sungkyunkwan University School of Medicine, Seoul, South Korea. We retrospectively evaluated 90 patients with multilobar epilepsy who underwent resection surgery between May 1995 and October 2013. Participants had iEEG monitoring to localize the epileptogenic foci and to determine the resection margin. Epilepsy was intractable before surgery despite proper and sufficient antiepileptic drug (AED) treatment. Clinical characteristics registered for each patient included age of seizure onset, age at surgery, duration of epilepsy, history of febrile seizures, monthly seizure frequency, and number of AEDs at surgery.

### Ethics Statement

All patients provided written informed consent for their participation in the study. Written informed consent was obtained from the next of kin, caretakers, or guardians on the behalf of the minors/children participants involved in this study. The study was approved by Institutional Review Board of Samsung Medical Center.

### Presurgical evaluation

Intractable epilepsy patients received a comprehensive pre-surgical evaluation consisting of complete neurologic examination, scalp video-electroencephalography (EEG) monitoring and brain magnetic resonance imaging (MRI) during the first admission period. Ictal and inter-ictal SPECT studies were performed to lateralize or localize epileptic foci. In case of multilobar epilepsy, the patient underwent PET and neuropsychological tests during the second admission. All data from these admissions were reviewed and discussed in an epilepsy management conference and the surgical strategy, including intracranial EEG (iEEG) monitoring, was established to remove the epileptic foci. In this study, multilobar epilepsy was defined when multilobar surgical resection was performed after multiple ictal onset zones were confirmed from more than two lobes during the iEEG monitoring.

### Analyses of clinical seizures during scalp EEG monitoring

We reviewed each patient’s seizures carefully. The presence of aura was determined by patient memory or the patient pressing a button before seizures.

### Scalp video EEG monitoring

The 10/10 system for scalp electrodes was used. AEDs were usually reduced or stopped to facilitate the recording of seizures.

- *Interictal EEG classification*: Interictal epileptiform discharges (IED) were defined as *unilobar* when 75% or more preponderance in one lobe and as *multilobar* when over ≥2 lobes with less than 75% preponderance in any single lobe.
- *Ictal EEG classification*: *Unilobar* was defined when the location of scalp ictal EEG onset (sEEG onset) was confined to one lobe, the amplitude ratio of one lobe versus the other lobes was greater than 2:1 in bipolar montages, and greater than 2:1 for the two sides in referential montages. *Multilobar* was defined when the sEEG onset initiated from ≥2 lobes over both hemispheres independently or synchronously.

### iEEG monitoring

iEEG was performed using a combination of grids/strips or depth electrodes. Anatomical targeting of electrodes was established in each patient according to available non-invasive information and hypotheses regarding localization of the epileptogenic zone. Concomitant implantation of depth electrodes was performed in nine patients (temporo-occipital [TO], n = 3; fronto-temporal [FT], n = 4; fronto-temporo-parietal [FTP], n = 1; temporo-parieto-occipital [TPO] lobe epilepsies, n = 1). Depth electrodes were inserted uni- or bilaterally into the mesial temporal region (amygdala and/or hippocampus) and the electrode locations were confirmed by intraoperative photographs and post-implantation surface-rendered electrode images. Ictal onset zone (IOZ) was defined when any paroxysmal, sustained ictal EEG pattern during the iEEG monitoring was distinct from background activity and accompanied by clinical seizures.[16] The detailed classification among multilobar epilepsies was determined through the iEEG monitoring of the IOZ site during at least three seizures, regardless of brain lesions.

### Neuroimaging studies

#### Brain MRI

MRI was performed using a GE Signa 1.5-Tesla scanner (GE Medical Systems, Inc., Milwaukee, WI, USA) or a 3.0-Tesla scanner (Philips, Best, the Netherlands). All patients underwent Spoiled Gradient Echo, T2-weighted and Fluid Attenuated Inversion Recovery imaging protocols. Also, MRI results were classified as lesional or non-lesional according to the presence of visible lesions with potential epileptogenicity.

#### FDG-PET studies

PET images were obtained (GE Advance PET scanner, GE Medical Systems, Inc.) after patients had fasted for four or more hours followed by intravenous injection of 7–10 mCi (259–370 MBq) of FDG. EEG during the uptake period demonstrated no EEG seizure activity in any patient. Hypometabolism was determined semi-quantitatively by visual assessment using calibrated color scales. A graduated color scale in 2% increments was used for display and analysis. When the metabolism of the lobe showed a 20% or more reduction compared with the other areas of metabolism, it was regarded as abnormal hypometabolism. [17]

#### Interictal and ictal SPECT studies

Brain SPECT scans were performed 30–60 min after injection of 25 mCi 99mTc-ethyl cysteinate dimer (ECD) using a 3-headed Triad XLT system (Trionix Research Laboratory, Inc., Twinsburg, OH). Interictal SPECT studies were performed when the patients had no documented seizure activity for 24 hours. For ictal studies, patients received radiotracer injections during seizures. The mean radiotracer injection time during the ictal SPECT was 30.0 ± 14.8 seconds (range, 14–105) and the mean seizure duration during injection was 85.8 ± 36.5 seconds (range, 18–190). Ictal-Interictal SPECT Subtraction Co-registered to MR images (SISCOM) analysis was performed on an offline workstation with ANALYZE 7.5 software (Biomedical Imaging Resource, Mayo Foundation, Rochester, MN) and as previously described.[18]

If hyperperfused areas include the IOZ, it is defined as localized. Non-localized indicated hyperperfused spots that were not found within the IOZ. Localized PET was defined when the IOZ was included in the hypometabolic areas, whereas, non-localized PET was when the IOZ was not involved in the hypometabolic areas.

Two neurologists (Cho EB and Joo EY) reviewed the neuroimaging results independently without patient information. In case of discrepancies, the final decision was made by formal reports from the Departments of Radiology or Nuclear Medicine.

### Surgery and outcomes

Complete resection was defined as when resection margins include the IOZ with or without frequent interictal spikes in adjacent brain regions and early ictal propagation on the iEEG monitoring. All patients had a minimum follow-up period of 1 year, up to 19 years. When the IOZ was diffuse (non-localized), or included eloquent areas within the resection margins, incomplete resection was performed. Patients were classified as “seizure-free” if they achieved Engel Class I by the last year of follow-up and “completely seizure-free” no seizures occurred after surgery. Early seizures were defined when seizures occurred within 6 months after surgery. The patients were instructed to visit the clinic 1 month after surgery, then every 3 months. If patients became seizure-free, they visited the clinic every 6 months. Postoperative seizure frequency and any possible provocative factors were documented. Surgical outcomes were evaluated at 6 months, 1, 2, and 5 years and at the end of the study period. Postoperative seizure outcomes were determined by outpatient clinic or telephone interviews using Engel’s classification.

### Statistical analyses

For comparison between seizure-free (Engel I) patients and patients with recurrent seizures (Engel II-IV), Chi-square or Fisher’s exact test were applied for categorical variables. A Student’s *t* test or Mann-Whitney *U* test was performed for continuous variables. Logistic regression analyses were used to verify independent risk factors for seizure recurrence. Variables with *p* values ≤ 0.05 in the simple logistic regression were tested for multiple logistic regression analysis. Statistical significance was accepted at *p* < 0.05. The time to first seizure recurrence was plotted using a Kaplan-Meier survival curve to estimate the proportion of individuals remaining seizure-free at various time points, according to several prognostic factors. A log rank test and a comparison of 95% confidence intervals were used to establish differences between good and bad prognostic factors of seizure recurrence after surgery.

## Results

### Patient characteristics

Demographics and clinical features of the 90 patients are summarized in Table 1. The results of scalp EEG monitoring and neuroimaging were compared between good (Engel I, n = 46) and poor surgical outcomes (II-IV, n = 44) (Table 2). Interictal PET was done in 79 patients (79/90, 87.7%) and SISCOM with interictal and ictal SPECT was performed in 75 patients (75/90, 83.3%). Seventy patients (70/90, 78.0%) underwent both PET and SPECT studies. Localized PET or SISCOM results were more frequently observed in patients with good surgical outcomes (Engel I). Both localized PET and SISCOM results were found in 26 patients and all had good surgical outcomes (Engel I). During the ictal SPECT, the proportion of patients with injection time ≤30 sec from seizure onset was not different between the localized and the non-localized groups (63.3% vs. 68.8%, *p* = 0.652). A considerable number of patients from both groups had lesional MRIs (76.1% in Engel I and 70.5% in Engel II-IV) without a statistical difference (*p* = 0.054).

**Table 1:** Patient characteristics.

	N = 90
**Demographics**	
Female (%)	57 (63.3)
Age at seizure onset, year	12.7 ± 8.8
Age at surgery, year	25.9 ± 10.6
Epilepsy history before surgery, year	14.2 ± 8.6
Follow-up period after surgery, year	8.0 ± 5.4
**Clinical features**	
Auras (%)	63 (70.0)
Seizure frequency per month (median [IQR])	2.5 (1.2–4.6)
GTC seizures (%)	77 (85.6)
Number of AEDs before surgery	3 ± 1
History of febrile seizure (%)	8 (8.9)
Family history of epilepsy (%)	4 (4.4)
**Ictal onset zone (IOZ)** *	
FT (%)	30 (33.3)
FP (%)	8 (8.9)
PT (%)	18 (20.0)
TO (%)	26 (28.9)
PO, FTO, TPO (%)	2 (2.2), 2 (2.2), 2 (2.2)
FO, FTP (%)	1 (1.1), 1 (1.1)

Continuous variables are presented as mean ± SD unless otherwise indicated. GTC, generalized tonic-clonic; AED, antiepileptic drugs; FT, frontotemporal; FP, frontoparietal; PT, parietotemporal; TO, temporooccipital; TPO, temporoparietooccipital; FTO, frontotemporoocipital; FO, frontooccipital; PO, parietooccipital; FTP, frontotemporooccipital.

*ictal onset zone (IOZ) identified during intracranial EEG monitoring

**Table 2:** Preoperative evaluations according to seizure outcomes in the last year of follow-up.

	Engel I (n = 46)	Engel II-V (n = 44)	P value
**Scalp IED**			0.371
Unilobar	21 (45.7)	16 (36.4)	
Multilobar	25 (54.3)	28 (63.6)	
**Scalp EEG onset**			0.159
Unilobar	13 (28.3)	7 (15.9)	
Multilobar	33 (71.7)	37 (84.1)	
**Brain MRI**			0.546
Lesional	35 (76.1)	31 (70.5)	
Non-lesional	11 (23.9)	13 (29.5)	
^**#**^ **PET**, n = 79			0.018*
Localized	26/40 (65.0)	15/39 (38.5)	
Non-localized	14/40 (35.0)	24/39 (61.5)	
^#^ **SISCOM**, n = 75			0.015*
Localized	25/39 (65.8)	14/36 (37.8)	
Non-localized	14/39 (34.2)	22/36 (62.2)	

Continuous variables are presented as mean ± SD; categorical variables are presented as N (%). IED, interictal epileptiform discharges; IOZ, ictal onset zone; MRI, magnetic resonance imaging; PET, positron emission tomography; SISCOM, subtraction ictal SPECT co-registered with MRI.

^#^PET/SISCOM, Localized/non-localized are determined by ictal onset zone during the intracranial EEG monitoring.

*p < 0.05, Chi-Square test

### Multilobar resection and postoperative findings

Fifty-five patients (61.1%) underwent left hemisphere resection and 35 (38.9%) underwent right-sided resection. Complete resection, as determined by IOZ on iEEG, was achieved in 64 patients (71.1%). Of them, 60.9% remained seizure-free (Engel I, 39/64), while 39.1% suffered persistent seizures after surgery (Engel II-IV, 25/64). Of 26 patients with incomplete resection, 19.2% remained seizure-free (Engel I, 5/26) and 80.8% did not (Engel II-IV, 21/26). Surgery of FT, parieto-temporal (PT) or TO constituted 81.1% of the resection types (73/90). Seven patients underwent multiple resections over three lobes (FTP, n = 3; TPO, n = 2; fronto-temporo-occipital [FTO], n = 2) in a single hemisphere. Complete lesion resection was achieved in 68% of patients (45/66) with lesional MRI findings.

There were complications in 14 (15.5%) of the patients: 4 of them (4.4%) had a persistent paresis of Medical Research Council (MRC) grade 4−4+, 4 (4.4%) had visual field defects, 4 (4.4%) had mild language disturbance such as naming difficulty, 3 (3.3%) had surgical site infection that needed drainage, and 1 (1.1%) had mild difficulty in face recognition.

Pathologic findings consisted of cortical dysplasia (50/90, 55.6%), hippocampal sclerosis (10/90, 11.1%), gliosis (9/90, 10%), tumors (8/90, 8.9%; ganglioglioma, n = 2; astrocytoma, n = 2; dysembryoplastic neuroepithelial tumors, n = 2; glioneuronal tumor, n = 1; and Sturge-Weber syndrome, n = 1), tuberous sclerosis (4/90, 4.4%) and other pathologies (9/90, 10%; parasites, n = 3; anomalous vessels, n = 2; cerebral infarcts, n = 2; fibrocalcific nodule, n = 1; and nonspecific, n = 1).

Postoperative scalp EEG was performed 6 months post-operation. Interictal spikes were found in 38.8% of patients (35/90).

### Postoperative progress and outcomes

The median postoperative follow-up time was 7.3 years (interquartile range, 2.9–12.6) and approximately 77% of patients completed a 5-year follow-up. In the first year after surgery, 56 (62.2%) patients were seizure-free (Engel I), and 47 patients (47/90, 52.2%) remained seizure-free at the last follow-up. Of the Engel class IV (19/20) patients at 1 year after surgery, 95% had persisting seizures at the last follow-up. Table 3 depicts seizure outcomes in each group.

**Table 3:** Engel classification of postoperative outcomes.

	1^st^ year (%)	2^nd^ year (%)	5^th^ year (%)
	N = 90	N = 79	N = 69
Engel I	56 (62.2)	42 (53.2)	32 (46.4)
Engel II	6 (6.7)	9 (11.4)	17 (24.6)
Engel III	8 (8.9)	10 (12.7)	4 (5.8)
Engel IV	20 (22.2)	18 (22.8)	16 (23.2)

**Fig 1:**
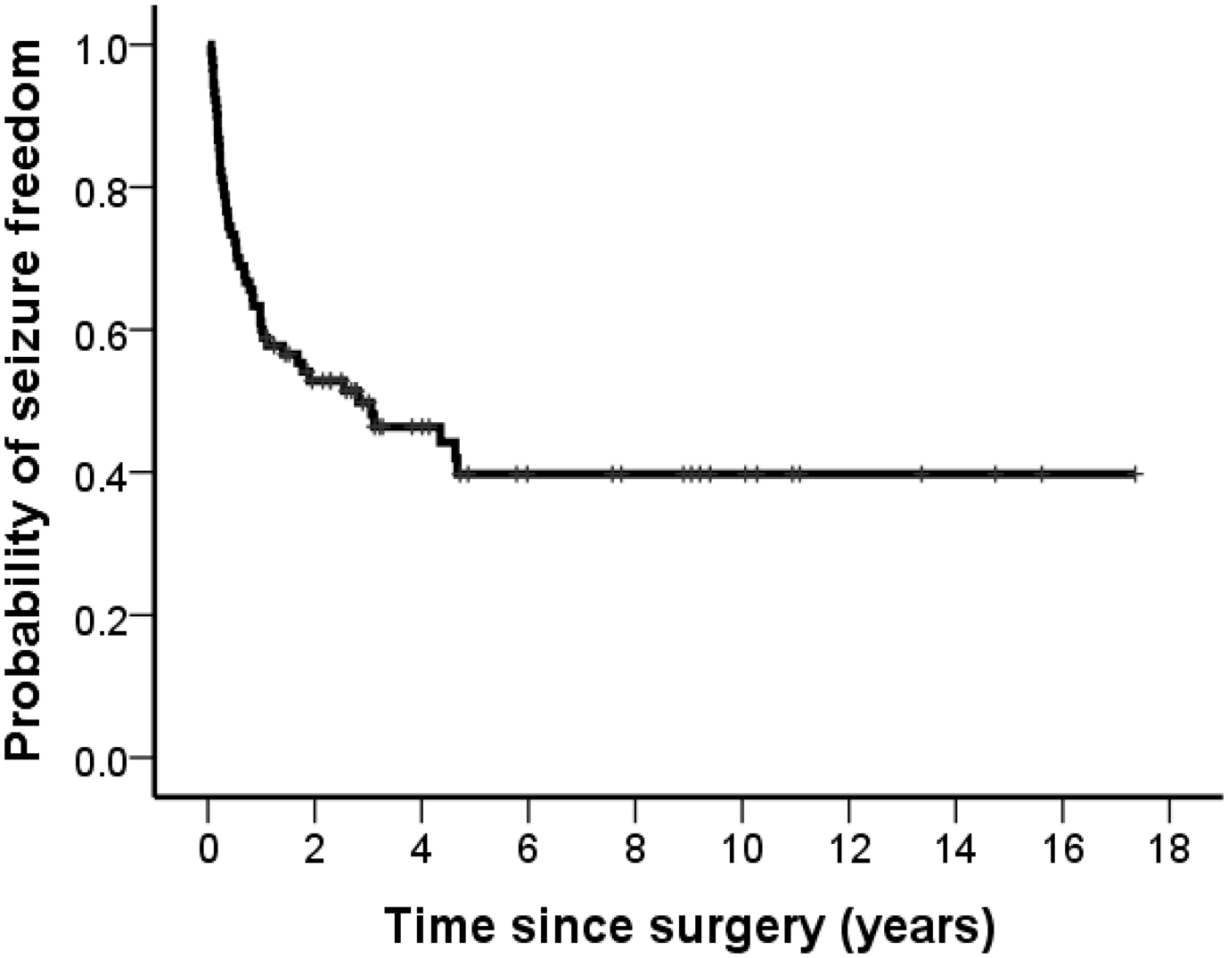
A Kaplan-Meier plot of time to the first seizure. Of 90 patients who underwent multilobar epilepsy surgery, 50 had a recurrence of seizures. 72% (n = 65) of patients were seizure-free at 6 months, 60% (n = 54) at 1 year, and 40% (n = 35) at 5 years.

A Kaplan-Meier plot of time to the first seizure after surgery revealed 72% (n = 65, 95% CI 63–82) of patients were seizure-free at 6 months, 60% (n = 54, 95% CI 50–70) at 1 year, and 40% (n = 35, 95% CI 28–52) at 5 years (Fig 1). Early seizures occurred within 6 months for 25 patients (50%). Another 25 patients (50%) showed seizure recurrence 6 months after surgery. However, the final outcomes of patients with early seizures were not different from patients with late seizure recurrence. In the last follow-up, the number of patients with early seizures was four for Engel I, 10 for Engel II, one for Engel III, and 10 for Engel IV. Similarly, the number of patients with late seizures was three for Engel I, nine for Engel II, four for Engel III, and nine for Engel IV.

Analysis based on resection type revealed that TO and PT resections had better outcomes. At 5 years, 55% of TO and 56% of PT resections were seizure-free, while 34% of FT and 14% of FP resections were seizure-free. In log rank tests, FP resections (n = 6, 85.7%) had more recurrent seizures than TO resections (n = 12, 46.2%) (*p* = 0.027).

There were no significant differences in seizure outcomes among patients with different pathologies. The percentage of patients with focal cortical dysplasia (57.4%, 31/54) that were seizure-free after 6 months, 1 year, and 5 years was 61% (95% CI 48–74), 52% (95% CI 38–66) and 36% (95% CI 21–51), respectively.

### Risk factors for seizure recurrence

**Fig 2:**
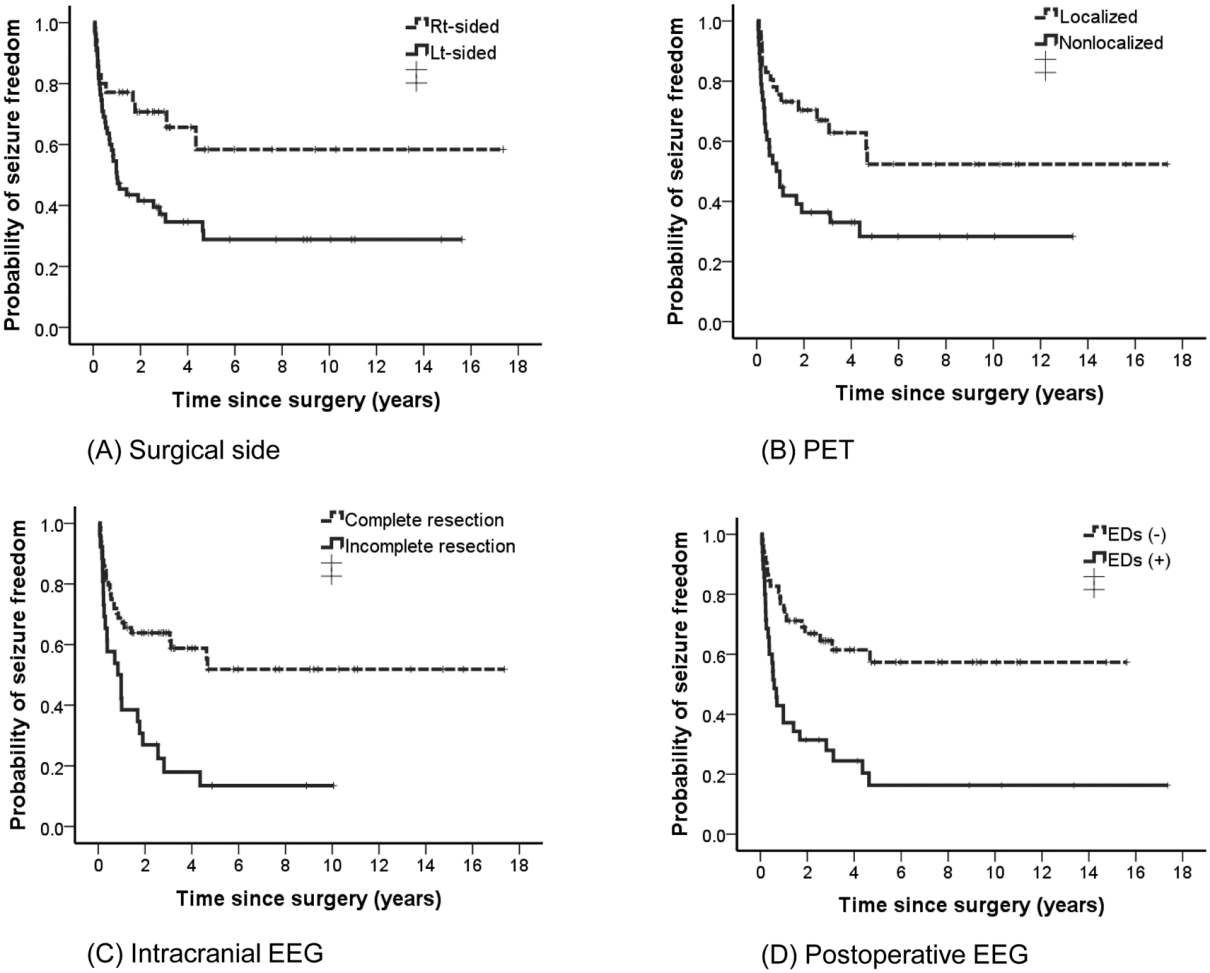
A Kaplan-Meier survival plots for groups with and without each predictor (A-D) from surgery to seizure recurrence. In the last year of follow-up, left-sided resection, non-localized PET, and incomplete resection were associated with recurrent seizures. PET, positron emission tomography; EEG, electroencephalography

Significant prognostic factors for seizure recurrence at 1-year post-operation were age at surgery, absence of aura, left-sided resection, non-lesional MRI, and non-localized PET (Table 4). In the last year of follow-up, left-sided resection, non-localized PET, and incomplete resection were associated with recurrent seizures (Table 5, Fig 2A–2C).

**Table 4:** Prognostic factors of seizure outcomes 1 year after surgery.

	Engel I (n = 55)	Engel II-IV (n = 35)	OR (95% CI)	*p*
Age at surgery, years	28 ± 10.6	22 ± 9.3	0.91 (0.85–0.98)	0.008
Auras absent	9 (16.4)	18 (51.4)	5.24 (1.45–18.94)	0.012
Left-sided resection	27 (49.0)	28 (80.0)	4.19 (1.15–15.29)	0.030
MRI, non-lesional	10 (17.9)	14 (38.2)	4.40 (1.18–16.37)	0.027
PET, non-localized	19/49 (40.0)	19/30 (63.3)	3.49 (1.02–11.99)	0.047

Continuous variables are presented as mean ± SD; categorical variables are presented as N (%). EDs = epileptiform discharges. Multiple logistic regression was performed using variables, such as age at surgery, the presence of aura, left-sided resection, MRI (lesional vs. non-lesional), hypometabolic areas on PET (localized vs. non-localized) and incomplete resection.

**Table 5:** Prognostic factors of seizure outcomes at the last follow-up.

	Engel I (n = 46)	Engel II-IV (n = 44)	OR (95% CI)	*p*
Left-sided resection	22 (47.8)	33 (75.0)	4.22 (1.42–12.58)	0.010
PET, non-localized	14/40 (35.0)	24/39 (61.5)	3.10 (1.05–9.12)	0.040
Incomplete resection	5 (10.9)	21 (47.7)	5.43 (1.61–18.35)	0.007

Categorical variables are presented as N (%). EDs = epileptiform discharges. Multiple logistic regression was performed using variables, such as Lt-sided resection, hypometabolic area on PET (localized vs. non-localized) and incomplete resection.

Postoperatively, the presence of interictal epileptiform discharges on scalp EEG were found to be a potent predictor of poor seizure outcomes in the last year of follow-up (OR = 3.62; 95% CI = 1.46–8.97; *p* = 0.005) (Fig 2D).

There was a significant correlation between incomplete resection and IED on postoperative scalp EEG (*p* = 0.001). Among 35 patients showing IED on postoperative EEG, 12 were seizure-free (Engel I) and 23 were not (Engel II-IV) in the last year of follow-up.

## Discussion

We observed long-term surgical outcomes in patients with multilobar epilepsy (median 7.4 years, 2.9–12.6) and found 40% complete seizure freedom (no postsurgical seizures) at 5 years after surgery. In the last year of follow-up, 52.2% of the 90 patients remained seizure-free (Engel I). This is comparable to a prior study demonstrating 41–44.4% seizure freedom after multilobar resection.[6] The discrepancy of seizure-free rates may result from different subtypes of populations (multilobar vs. extra-temporal, adults vs. pediatric or mixed subjects) and different surgical techniques. Follow-up of up to 19 years in this study provides a more appropriate prognosis of multilobar surgeries than a shorter follow-up. We found that patients with excellent outcomes 1 year after surgery might experience seizures within this follow-up. Most, however, had achieved seizure freedom by the end of follow-up (47/55, 85.4%). Similarly, patients with the worst outcomes (Engel IV) still had persistent seizures in the end (19/20, 95%). Engel II patients showed the most fluctuant course, however, their seizures finally decreased in the last year of follow-up (6.7% → 21.1%).

Over half of patients (50/90, 55.5%) experienced at least one seizure after surgery. Early seizures occurred in 25 patients (25/50, 50%), similar to previous studies.[7,19] However, final outcome of patients with early seizures did not differ from patients with late seizures. Fourteen patients (14/25, 56%) with early seizures had favorable outcomes (Engel I, n = 4; Engel II, n = 10), while the rest (11/25, 44%) had unfavorable outcomes (Engel III, n = 1; Engel IV, n = 10) in the last year of follow-up. Interestingly, the final outcome of patients with early seizures was not different in 25 patients with seizure recurrence after 6 months (Favorable: 12/25, 48% [Engel I, n = 3; Engel II, n = 9] vs. unfavorable: 13/25, 52% [Engel III, n = 4; Engel II, n = 9]). It was interesting that the surgical patients who experienced early seizures had no difference in outcomes compared to those with late seizures. Sarkis et al. suggested [7] that incomplete resection of epileptic foci might result in seizure recurrence. However, we did not find any differences of resection margin including IOZ, PET/SPECT findings (localized/non-localized in relation to IOZ), or other clinical features between patients with early seizures and those with late seizures in this study. Meanwhile, we found the changes of AEDs in patients with favorable outcome. Mean number of AEDs were increased in patients with favorable outcome (2.4 → 4.1), but not in patients with poor outcome. Inadequate treatment due to vulnerability to high-dose medications or side effects from multiple AEDs may result in dissatisfied outcome.

Non-localized FDG-PET was a significant prognostic factor for patients with poor outcomes in both the first and the last year of follow-up. To the best of our knowledge, this is the first study presenting a prognostic role for PET findings before multilobar resection. It suggests that localized hypometabolic findings on PET are closely associated with good surgical outcomes. PET reveals interictal brain dysfunction with decreased metabolic need can possibly detects invisible pathologic tissues.[19,20] PET is widely utilized for cryptogenic neocortical epilepsy surgery [12,21,22] and good surgical outcomes were reported after resection of localized hypometabolic areas in several surgical studies.[11,22,23] This means that concordantly localized hypometabolism on PET guides the insertion of subdural grids with confidence and leads to complete resection of epileptic foci, which applies to cases with multilobar epilepsy. Sarkis et al. also reviewed PET for presurgical evaluation. However, they classified findings as normal versus abnormal (ipsilateral vs. bilateral), which showed no differences.[7] Yu et al. reviewed head-PET-computed tomography records of patients with uni- or multilobar epilepsies, but they did not evaluate the role of imaging in prognosis in detail.[6] In this study, we classified PET findings as localized or non-localized according to the IOZ on scalp EEG monitoring. This process is routinely performed during presurgical evaluation in our epilepsy clinic and the results guide surgical planning for patients. We try to cover hypometabolic lesions as much as possible with subdural grids or strips. Mismatches between IOZ and hypometabolic zones (i.e. non-localized PET) may result in mislocation of subdural electrodes or incomplete coverage of epileptic foci, leading to poor surgical outcomes.

Another functional neuroimaging technique, the SPECT (SISCOM) alone, was not a predictive factor during the first or last year of follow-up, contradicting previous reports.[11,24,25] In the whole, we confirmed that patients with both localized PET and SISCOM (n = 26) had good surgical outcomes for multilobar epilepsy in the last year of follow-up (Engel I). Non-lesional MRI was correlated with seizure recurrence at 1-year follow-up, although it was not a potent predictor for poor outcomes in the last year. Rates of good surgical outcomes for non-lesional epilepsy ranged from 26 to 66% according to surgical location, with extra-temporal lobe epilepsy being less effective (particularly for frontal and parietal lobes).[19–21,26] Eleven of 24 patients with non-lesional MRI (11/24, 45.8%) showed good seizure outcomes (Engel I) and all showed localized PET and SISCOM. This supports the complementary roles of functional neuroimaging to achieve good surgical outcomes in patients with cryptogenic multilobar LRE.

Seizure recurrence was more frequent in patients with left-sided resection than right-sided resection. The location and laterality of surgery may be associated with surgical outcomes.[2,3,27,28] Surgical outcomes of left-sided frontal lobe epilepsy are much worse than right-sided,[26,27] which is explained by a higher frequency of incomplete resection (left vs. right; 30.8% vs. 0%; *p* = 0.039). Since most patients are right-handed, left-sided resection may be limited by eloquent areas (language areas in the dominant hemisphere). This is congruent with the finding that incomplete resection of epileptic foci was an independently poor prognostic factor in the last year of follow-up and FP resection had the worst surgical prognosis.

In previous studies, postoperative IEDs on EEG were used as a prognostic factor for seizure recurrence.[7,29,30] Also, we were able to reproduce these results and found a correlation between IED spikes and incomplete resection of IOZ (*p* = 0.01), suggesting residual epileptogenic tissue. [7]

In conclusion, we observed surgical outcomes in patients with multilobar epilepsy during long-term follow-up. Functional neuroimaging, PET and SISCOM may be potent prognostic factors for postoperative outcomes following multilobar resection in patients with LRE.
